# Inactivation of the β(1,2)-xylosyltransferase and the α(1,3)-fucosyltransferase genes in *Nicotiana tabacum* BY-2 Cells by a Multiplex CRISPR/Cas9 Strategy Results in Glycoproteins without Plant-Specific Glycans

**DOI:** 10.3389/fpls.2017.00403

**Published:** 2017-03-27

**Authors:** Sébastien Mercx, Nicolas Smargiasso, François Chaumont, Edwin De Pauw, Marc Boutry, Catherine Navarre

**Affiliations:** ^1^Institut des Sciences de la Vie, Université catholique de LouvainLouvain-la-Neuve, Belgium; ^2^Mass Spectrometry Laboratory, Molecular Systems Research Unit, Université de LiègeLiège, Belgium

**Keywords:** antibody, plant-specific glycans, humanized *N*-glycosylation, molecular farming, gene editing, glycoproteins, glyco-engineering, suspension cells

## Abstract

Plants or plant cells can be used to produce pharmacological glycoproteins such as antibodies or vaccines. However these proteins carry *N*-glycans with plant-typical residues [β(1,2)-xylose and core α(1,3)-fucose], which can greatly impact the immunogenicity, allergenicity, or activity of the protein. Two enzymes are responsible for the addition of plant-specific glycans: β(1,2)-xylosyltransferase (XylT) and α(1,3)-fucosyltransferase (FucT). Our aim consisted of knocking-out two *XylT* genes and four *FucT* genes (12 alleles altogether) in *Nicotiana tabacum* BY-2 suspension cells using CRISPR/Cas9. Three *XylT* and six *FucT* sgRNAs were designed to target conserved regions. After transformation of *N. tabacum* BY-2 cells with genes coding for sgRNAs, Cas9, and a selectable marker (*bar*), transgenic lines were obtained and their extracellular as well as intracellular protein complements were analyzed by Western blotting using antibodies recognizing β(1,2)-xylose and α(1,3)-fucose. Three lines showed a strong reduction of β(1,2)-xylose and α(1,3)-fucose, while two lines were completely devoid of them, indicating complete gene inactivation. The absence of these carbohydrates was confirmed by mass spectrometry analysis of the extracellular proteins. PCR amplification and sequencing of the targeted region indicated small INDEL and/or deletions between the target sites. The KO lines did not show any particular morphology and grew as the wild-type. One KO line was transformed with genes encoding a human IgG2 antibody. The IgG2 expression level was as high as in a control transformant which had not been glycoengineered. The IgG glycosylation profile determined by mass spectrometry confirmed that no β(1,2)-xylose or α(1,3)-fucose were present on the glycosylation moiety and that the dominant glycoform was the GnGn structure. These data represent an important step toward humanizing the glycosylation of pharmacological proteins expressed in *N. tabacum* BY-2 cells.

## Introduction

The production of recombinant proteins in plants (molecular farming) is seen as an interesting alternative to the current means of production of biopharmaceutics in microbial and mammalian cultures. Plants have the advantages of higher eukaryotes (complex protein folding and post-translational modifications). Although whole plants have been used intensively (especially transient expression in *Nicotiana benthamiana* leaves) to produce recombinant proteins, the molecular farming field is very diverse and many different production platforms exist (Makhzoum et al., 2014). One of them is based on plant suspension cells. The first example of the production of a recombinant protein (human albumin) in plant suspension cells dates back to Sijmons et al. (1990). Plant cell cultures require a simple and inexpensive growth medium and proteins such as antibodies can be secreted in the extracellular medium, facilitating the purification step. Moreover, plant cells are grown in a contained bioreactor avoiding the regulatory issues encountered by whole plant production (Santos et al., 2016).

Plants can perform the *N*-glycosylation of proteins similarly to mammalian cells but there are still regulatory issues regarding the differences between plant and mammalian glycans. Indeed plants carry core β(1,2)-xylose and core α(1,3)-fucose that are not present in mammalian glycoproteins. As a consequence, those residues can possibly elicit an immune or even allergic response. While the immunogenicity of these residues has been demonstrated in different studies (Wilson et al., 1998; van Ree et al., 2000; Altmann, 2007; Bosch et al., 2013), others did not observe any adverse immune response of the injected recombinant protein such as the plant-produced taliglucerase and the influenza virus-like particles (Grabowski et al., 2014; Ward et al., 2014). Thus, whether the plant glycans contribute to elicit an immune response or not is currently unclear. However, for safety and therapeutic efficiency reasons the immunogenicity needs to be checked for each particular pharmacological protein during the clinical trial. A plant-based expression platform expressing recombinant proteins devoid of any β(1,2)-xylose or α(1,3)-fucose would be therefore very welcome. Moreover, it would make the glycosylation more homogenous and potentially more efficient. For example, it has been shown that an antibody lacking xylose and fucose produced in silenced *N. benthamiana* and carrying β(1,4)-galactose outperformed the same antibody produced in CHO in a virus neutralization assay (Strasser et al., 2009).

Two enzymes are responsible for the addition of the plant-specific glycans: β(1,2)-xylosyltransferase (XylT) and α(1,3)-fucosyltransferase (FucT) (Strasser et al., 2004). Attempts were made to remove the plant type glycans by inactivating those two enzymes. A *XylT* and *FucT* knock out mutant has been reported in *Arabidopsis* plants (obtained by T-DNA insertion and crossing) (Strasser et al., 2004) as well as in the moss *Physcomitrella patens* (obtained by homologous recombination) (Huether et al., 2005). RNAi was used to downregulate *XylT* and *FucT* in *N. benthamiana* (Strasser et al., 2008), *Lemna minor* (Cox et al., 2006), and *Medicago sativa* (Sourrouille et al., 2008) plants, as well as in rice (Shin et al., 2011) and *Nicotiana tabacum* BY-2 cell lines (Yin et al., 2011). However, the RNAi approach has a major drawback: inactivation of the expression is never complete. A genome editing tool to precisely mutate any gene would be more appropriate. Before the discovery of CRISPR/Cas9 and its high potential to edit any given gene, there were three classes of sequence-specific nucleases used to inactivate genes in plants: the meganucleases, zinc-finger nucleases (ZFNs), and transcription activator-like effector nucleases (TALENs) (Voytas, 2013). Those technologies are not straightforward, especially when multiple genes must be inactivated. A TALEN approach was used very recently in order to knock-out the *XylT* and *FucT* genes in *N. benthamiana* plants (Li et al., 2016). Once again, although significant reduction of β(1,2)-xylose- and core α(1,3)-fucose was observed, complete loss of both enzymes was not achieved because not all of the isoforms were targeted.

CRISPR/Cas9 is a new type of sequence-specific nuclease. It has been shown to be very powerful, versatile, and able to inactivate multiple genes at the same time (Xie et al., 2015). Recently, we have shown that the CRISPR/Cas9 nuclease could be used to inactivate a gene in *N. tabacum* BY-2 cells (Mercx et al., 2016). In this study, we identified two *XylT* and four *FucT* genes (12 alleles) and successfully knocked-out these alleles by targeting conserved regions with CRISPR/Cas9. No trace of β(1,2)-xylose or α(1,3)-fucose could be detected by Western blotting or mass spectrometry. A knock-out line was further transformed for expressing an antibody. These data show that *N. tabacum* BY-2 cells can be engineered to humanize pharmacological glycoproteins produced in this host.

## Materials and Methods

### Plant Cell Cultures

*Nicotiana tabacum* cv. Bright yellow 2 (BY-2) (Nagata et al., 1992) suspension cells were grown in the dark at 25°C with agitation on a rotary shaker (90 rpm) in liquid MS medium [4.4 g/L Murashige and Skoog salts (MP BIOMEDICALS, Solon, OH), 30 g/L sucrose, 0.2 g/L KH_2_PO_4_, 2.5 mg/L thiamine, 50 mg/ml myo-inositol, and 0.2 mg/L 2,4-D, pH 5.8 (KOH)]. Cultures were grown in 50 mL of medium in a 250 mL Erlenmeyer flask and a 5% inoculum was transferred each week into fresh medium. Transformed cells were grown on solid medium supplemented with 15 μg/mL of bialaphos.

### *XylT* and *FucT* Gene Accessions

Genbank accessions are as follows: *XylTA* (NM_001324669), *XylTB* (NM_001325611), *FucTA* (XM_016657530), *FucTB* (XM_016620229), *FucTC* (NM_001324945), *FucTD* (XM_016585847).

### Cas9 and sgRNA Plasmid Construction and Plant Cell Transformation

The polycistronic tRNA-gRNA was synthesized (Genescript) and introduced into a pUC57 vector at the SbfI restriction site. The polycistronic sequence was then transferred into the SbfI cloning site of the pFGC-pcoCas9 binary vector (Li et al., 2013). The vector was transferred into *Agrobacterium tumefaciens* LBA4404virG (van der Fits et al., 2000) by electroporation. Transformation of *N. tabacum* BY-2 cells was performed as indicated in Mercx et al. (2016). The transgenic KO line 11 (see Results) was further transformed with the binary vector (pPZP-RCS2-nptII-mCherry-HIgG2-LoBM2) designed for the production of a human IgG2 antibody (Mercx et al., 2016).

### Analysis of Genome Modifications

Genomic DNA was extracted from stable transgenic transformants after bialaphos selection. PCR was performed using primers (Supplementary Table S1) flanking the targeted region. The PCR products were electrophoresed on an ethidium bromide-stained agarose gel (3%). Bands were extracted, purified, and cloned into the pGEM-T-easy vector and sequenced.

### SDS-PAGE and Western Blotting Analysis of Proteins

For extracellular protein glycosylation analysis, 1 mL out of 4 mL of a 7-day BY-2 culture was filtered on three layers of Miracloth (Calbiochem) and 30 μL of the filtrate were analyzed by reducing SDS-PAGE. For extracellular IgG2 production analysis, 1 mL of a 7-day BY-2 culture in a 50-mL flask was filtered on three layers of Miracloth (Calbiochem) and 30 μL were analyzed by non-reducing SDS-PAGE. For the total cellular proteins, 100 mg of solid calli were transferred into a 2 mL Micro tube (Sarstedt) containing 0.5 g of glass beads (0.85–1.23 mm) and 800 μL of homogenization buffer (250 mM sorbitol, 60 mM Tris–HCl, 2 mM Na_2_EDTA, pH 8.0) supplemented with 5 mM DTT, 1 mM phenylmethylsulfonylfluoride (PMSF), and protease inhibitor cocktail (leupeptin, aprotinin, antipain, pepstain, and chymostain, each at 2 μg/mL). Cell grinding was performed for 3 × 40 s at 5000 rpm (PrecellysTm24 Control Device Bertin Technologies) with 2 min pauses on ice. The samples were centrifuged for 10 min at 10,000 *g* (Eppendorf 5417C). The protein concentration in the supernatant was quantified and 15 μg of proteins were analyzed by SDS-PAGE (4–20% polyacrylamide) and either stained with Coomassie Brilliant Blue G-250 (SERVA, Heidelberg, Germany) or transferred onto a PVDF membrane (Millipore, Billerica, MA, USA) for Western blotting analysis. The PVDF membrane was incubated with the primary antibodies against β(1,2)-xylose (monoclonal antibody, Agrisera AS07 267; dilution 1:5,000) or α(1,3)-fucose (monoclonal antibody, Agrisera AS07 268: dilution 1:10,000), and then with the secondary HRP-conjugated antibodies against rabbit IgG (dilution 1:10,000). The signals were quantified using a Kodak Image Station 4000R (Eastman Kodak company, Rochester, NY, USA).

### Mass Spectrometry Analysis of Total *N*-glycans of the Secreted Proteins

Fifty mL of a 7-day old BY-2 suspension culture was filtered on three layers of Miracloth (Calbiochem) and 10 mL aliquots of the filtrate were centrifuged (8,000 *g*, 30 min) and the supernatant was concentrated to 1 mL using Amicon^®^ Ultra-4 centrifugal filter units, MWCO 3 kDa (Millipore). The proteins were precipitated by salting-out using (NH_4_)_2_SO_4_ (35% w/w, incubated on ice for 30 min after complete dissolution). The samples were then centrifuged for 10 min at 16,000 *g* and the supernatant was discarded. The pellet was solubilized in 50 mM NH_4_HCO_3_ and the proteins were reduced (incubation with 10 mM DTT, 56°C, 40 min) and alkylated (20 mM iodoacetamide, 20°C, 30 min). Additional purification was then performed using a 2D Clean-up Kit (GE Healthcare). The proteins were resuspended in 50 mM NH_4_HCO_3_ and digested using trypsin (first incubation at 1/50 (w/w) for 16 h at 37°C followed by a second incubation with 1/100 additional trypsin for 3 h in 80% acetonitrile at 37°C). After heat inactivation of trypsin and solvent evaporation, the peptides were resuspended in citrate-phosphate buffer (48.3 mM citric acid, 103.3 mM Na_2_HPO_4_, pH 5) and the *N*-glycans were released using PNGase A (0.2 mU/100 μg, 24 h at 37°C). Digestion was stopped by the addition of trifluoroacetic acid (0.5%). The *N*-glycans were separated from the peptide using Sep-Pack C18 cartridges (Waters) using a 1-propanol/5% acetic acid system. The *N*-glycans were then desalted using a Glycoclean H-cartridge (Prozyme) and evaporated to dryness. Next, they were resuspended in labeling solution (750 mM NaBH_3_CN, 175 mM 2-aminobenzidine in DMSO/acetic acid at a 10:3 ratio) and incubated at 65°C for 2 h. Purification on a Glycoclean *S*-cartridge (Prozyme) was finally performed according to the manufacturer’s protocol and the *N*-glycans were evaporated to dryness.

Before mass spectrometry analysis, the glycans were resuspended in 10 μL 50% acetonitrile. One microlliter of sample was mixed on a MALDI plate with 1 μL of matrix (2,5 dihydroxybenzoic acid prepared at 20 mg/mL in water/acetonitrile 50/50, 0.1% formic acid) and allowed to dry. For every sample, 10 mass spectra were recorded on a MALDI-TOF instrument (Ultraflextreme, Bruker Daltonics) by adding 5000 laser shots.

The data were analyzed using FlexAnalysis 3.4 software (Bruker Daltonics). The intensities of the glycan peaks were normalized to the total glycan signal allowing relative proportions to be determined. The detected peaks were annotated using GlycoWorkbench 2.

### Purification of IgG

Eight × 50 mL of a 7-day old BY-2 suspension culture was filtered on three layers of Miracloth (Calbiochem) and the filtrate was centrifuged (8,000 *g*, 30 min). The supernatant was recovered, supplemented with 10% 1 M Tris-Cl pH 8.0 and incubated for 16 h at 4°C with one mL of Pierce^®^ Protein A Plus Agarose (Thermoscientific # 22812) previously washed three times with 10 mL of 0.1 M Tris-Cl pH 8.0. The sample was then filtered through a nylon membrane and poured onto a poly-prep^®^ chromatography column (Biorad # 731-1550). After washing with 10 mL of 0.1 M Tris-Cl pH 8.0, IgG were eluted with 8 × 500 μL 0.1 M glycin pH 3.0 and immediately supplemented with 10% 1 M Tris-Cl pH 8.0. Ten microlliter of each elution fraction were used for SDS-PAGE (8% acrylamide) analysis followed by colloidal blue staining.

### Mass Spectrometry Analysis of IgG Glycosylation

The IgG sample was submitted to proteolysis as described above and in Navarre et al. (2017). The resulting (glyco)peptides were separated by reverse-phase chromatography using UPLC (MClass, HSS T3 column, Waters) in one dimension with an increasing ratio of acetonitrile/water (5–40% for 70 min) at a 600 nL/min flow rate. It was coupled to a Hybrid Quadrupole-Orbitrap Mass Spectrometer (Q-Exactive Plus, Thermo Fisher Scientific, USA), programmed for data-dependent acquisition mode. Survey scans were acquired at 70,000 mass resolving power (full width at half maximum). An ion mass range from 400 to 1600 m/z was acquired in MS mode, and 3E6 ions were accumulated in the survey scans. Ion trap Higher energy Collision Dissociation fragmentations at NCE 28 were performed within 1.6 amu isolation windows and a dynamic exclusion was enabled for 10 s. MS/MS spectra were searched for typical oxonium ions (m/z 204.09 and 366.14) indicating the presence of glycopeptides. The presence of Y1 ion (m/z 1360.60 corresponding to EEQFN^∗^STFR where ^∗^ = HexNAc) was also checked to confirm the glycosylation site. For the assigned spectra, the composition of the glycopeptides was determined based on their precursor mass using the Glycomod tool (available at http://web.expasy.org/glycomod/) and their MS/MS spectra. Following that step, the relative abundance of the glycopeptides was determined by integration of MS1 chromatograms using Skyline 3.1.

## Results

**FIGURE 1:**
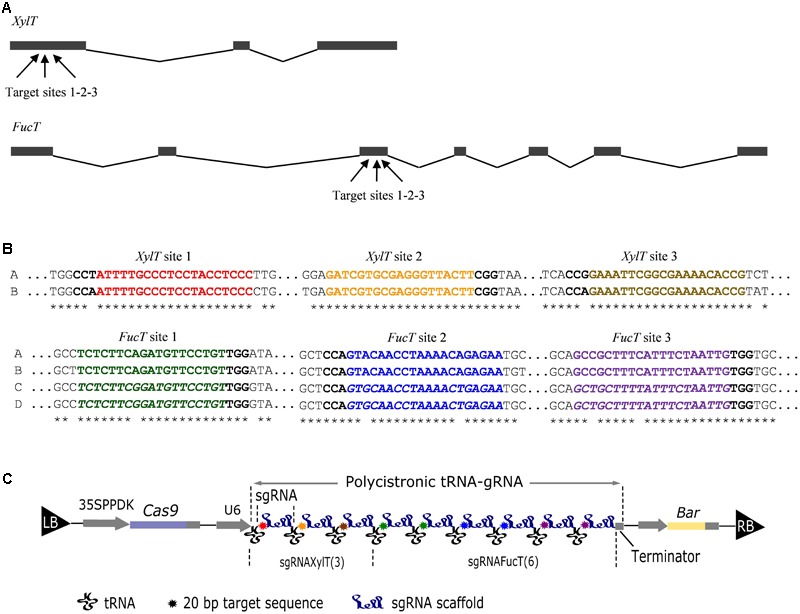
**DNA constructs aimed at inactivating the *XylT* and *FucT* genes. (A)** Schematic representation of the *XylT* and *FucT* genes. Black boxes and lines represent exons and introns, respectively. Arrows indicate the target sites for CRISPR-Cas9 as detailed in **(B)**. **(B)** Sequence of the *XylT* and *FucT* target sites for CRISPR-Cas9. The sequences of the two *XylT* (A,B) and the four *FucT* (A–D) genes is compared. Stars indicate conserved positions. The red, orange, and brown sequences correspond to the *XylT* sgRNAs. The green, blue, and violet sequences correspond to the *FucT* sgRNAs. For *FucT*, a divergent sequence is italicized. The PAM sequence is in bold. **(C)** Schematic representation of the binary vector pFGC-Cas9-tRNA-sgRNA_XylT(3)_–sgRNA_FucT(6)_. Left (LB) and right (RB) T-DNA borders are indicated. The *Cas9* gene is controlled by the 35S-PPDK transcriptional promoter. The synthetic polycistronic tRNA-gRNA consists of tandemly arrayed tRNA-gRNA units downstream of the U6 transcriptional promoter. A *bar* gene permits the selection of transformants.

In *N. tabacum* the *XylT* and the *FucT* genes have not been annotated yet. *N. tabacum* is an allotetraploid resulting from a cross between *Nicotiana sylvestris* and *Nicotiana tomentosiformis* and is thus expected to contain at least two sets of genes. We retrieved and aligned the coding sequences corresponding to putative *XylT* and *FucT* genes of three cultivars (TN90, BX, and K326) whose genomic sequences had been deposited on the Sol Genomics Network^1^ as well as on NCBI. For *XylT* we found two isoforms, one from *N. sylvestris* (*XylTA*: Ntab-TN90_AYMY-SS628) and one from *N. tomentosiformis* (*XylTB*: Ntab-TN90_AYMY-SS11650). For *FucT* we found four isoforms: two from *N. sylvestris* (*FucTA*: Ntab-TN90_AYMY-SS18207; *FucTD*: CDS-Ntab-TN90_AYMY-SS18127) and two from *N. tomentosiformis* (*FucTB*: Ntab-TN90_AYMY-SS18046; *FucTC*: Ntab-TN90_AYMY-SS15344). Genbank accession numbers for *XylT* and *FucT* mRNA annotations can be found in Section “Materials and Methods.” Unlike plants, suspension cells cannot be self-crossed to obtain homozygous mutants. Altogether, 12 target genes were selected for inactivation. To increase the likelihood of mutating all of them, we targeted the most conserved regions of the genes so that one sgRNA could target a maximum of isoforms. Therefore, three sgRNAs were designed to target three conserved regions of *XylT* exon 1 (where the two *XylT* genes are identical), and six sgRNAs were designed to target three conserved regions of *FucT* exon 3 (where the four genes group into two sequences) (**Figures 1A,B**).

**FIGURE 2:**
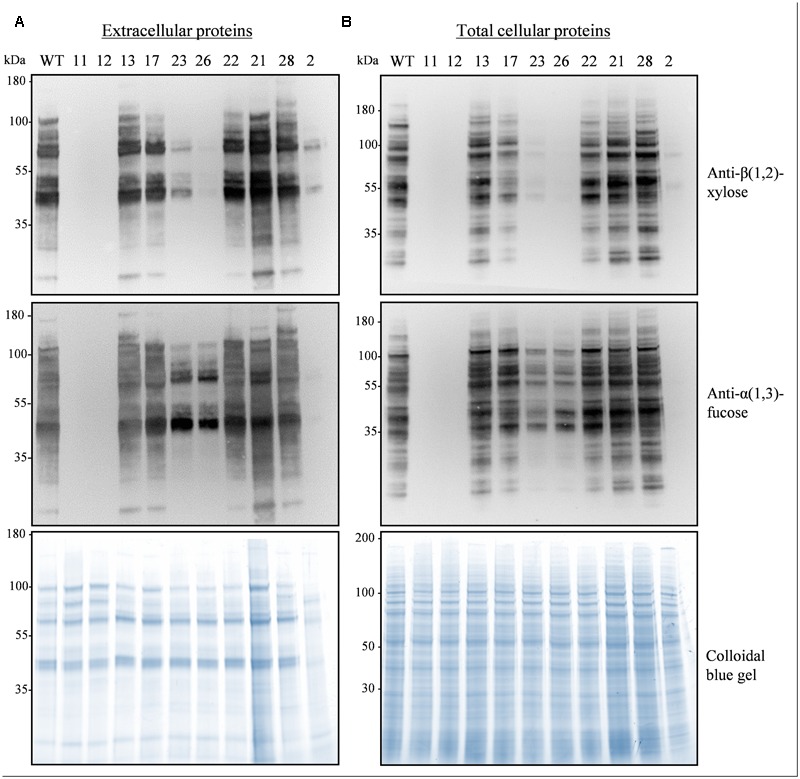
**Absence of α(1,3)-fucose and β(1,2)-xylose on glycoproteins from the *XylT* and *FucT* KO lines.** Secreted proteins (30 μl culture medium) **(A)** or total cellular proteins (15 μg) **(B)** from a WT and the indicated pFGC-Cas9-tRNA-sgRNA(9) transgenic lines were electrophoresed and analyzed by Western blotting using antibodies against β(1,2)-xylose and α(1,3)-fucose, A colloidal blue gel is displayed below as a loading control.

To assemble the sgRNA coding sequences we used the multiplex strategy in which a tRNA is positioned after each sgRNA and a U6 promoter is located upstream of the polycistron (Xie et al., 2015) (**Figure 1C**). The scaffold of the sgRNA was optimized to increase the targeting efficiency as proposed by Dang et al. (2015). The whole cassette was inserted into the pFGC-Cas9 binary vector containing the *Cas9* gene and a *bar* gene for selection (**Figure 1C**). After transformation of *N. tabacum* BY-2 cells with *A. tumefaciens* and selection on bialaphos, 28 transgenic lines were obtained and 10 were analyzed in more detail. The proteins secreted in the culture medium and the total cellular proteins were analyzed by Western blotting using antibodies recognizing β(1,2)-xylose and α(1,3)-fucose (**Figure 2**). Three lines (2, 23, 26) showed a strong reduction of β(1,2)-xylose and α(1,3)-fucose, while two lines (11, 12) were completely devoid of them, indicating complete gene inactivation.

MALDI-TOF MS analysis was performed to investigate the glycoform profile of the secreted proteins from lines 11 and 12. The structures of the total *N*-Glycans are presented in **Table 1**. The results confirm that no β(1,2)-xylose and no α(1,3)-fucose are present on the glycan moieties for lines 11 and 12, unlike the wild-type (WT) line in which more than 91% of the *N*-glycans carry a β(1,2)-xylose and more than 83% carry a α(1,3)-fucose residue. There is, however, one glycoform in the KO lines that carries a fucose (less than 0.8%), which most likely belongs to the Lewis epitope. Indeed, MS/MS analysis revealed a peak at 1222,794 m/z that corresponds to a Lewis epitope fragment (Supplementary Figure S2). In more detail (shown for line 11 and WT in Supplementary Figures S1–S3), the mass spectrum for lines 11 and 12 showed that GnM (41.1 and 39.1% of the total *N*-glycans, respectively) and GnGn structures (39.9 and 44.6% of the total *N*-glycans, respectively) are the most abundant and represent more than 80% of the total *N*-glycans. Several high-mannose type glycans are also present as well as the MM structure. In comparison, the WT line showed that the GnMXF, MMXF, GnGnXF structures (42.4%; 31.4%; 8.5% of the total *N*-glycans) are the most abundant glycoforms.

**Table 1:** Relative amount of total *N*-glycans (%) of the extracellular proteins of line 11, line 12 and Wt.

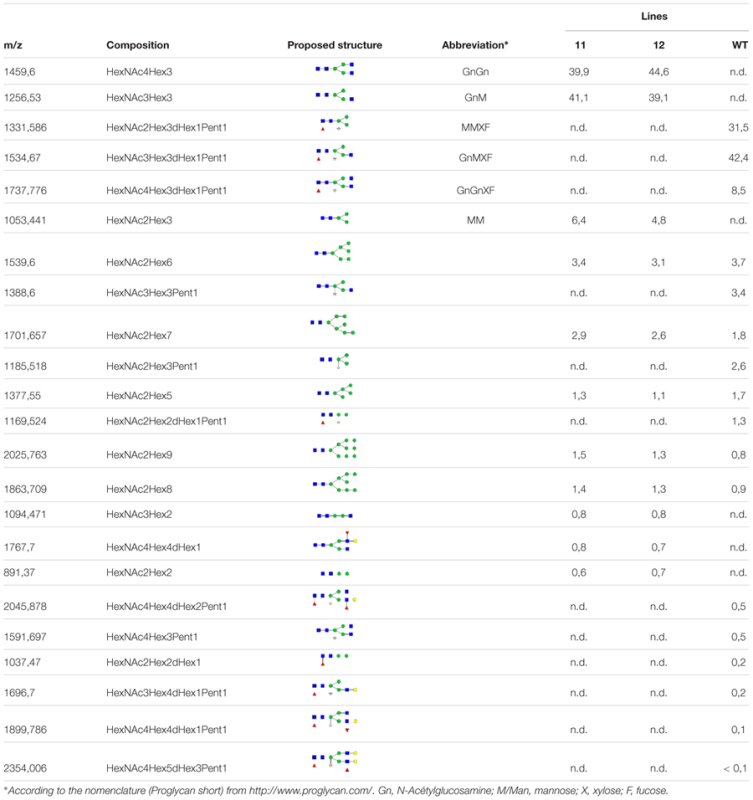

**FIGURE 3:**
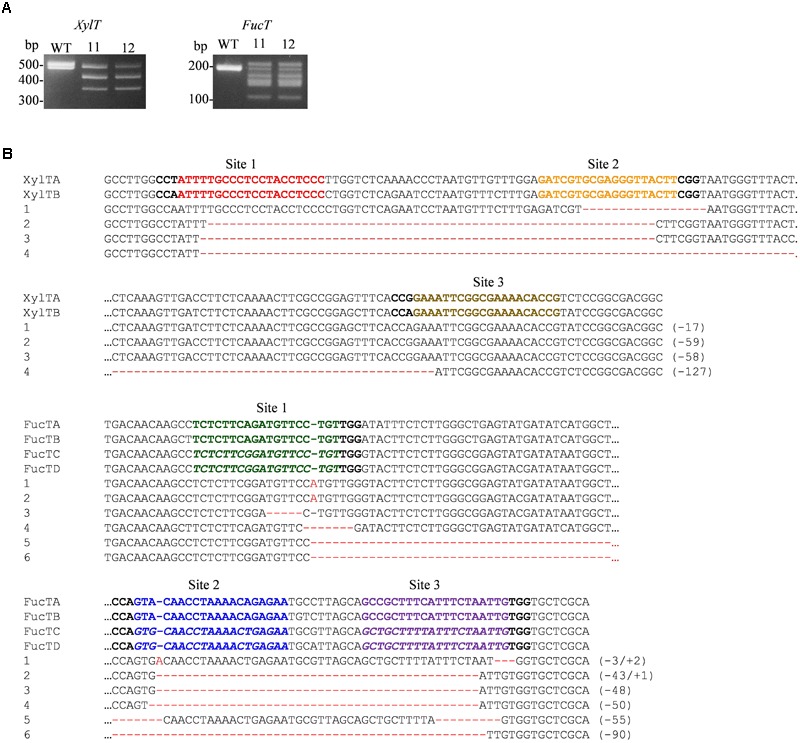
**(A)** PCR amplification of the *XylT* and *FucT* target regions. Genomic DNA prepared from the WT and the indicated transgenic lines was used to amplify the *XylT* and *FucT* target regions. **(B)** Sequences of the target regions in the PCR fragments. *XylTA, B* and *FucTA-D*: WT sequences; 1-6: sequences of line 11.

We sought to determine the modifications that occurred in the *FucT* and *XylT* loci. PCR amplification with primers hybridizing to conserved regions (Supplementary Table S1) of the *XylT* or *FucT* target region was performed. Electrophoresis analysis showed shortened fragments (**Figure 3A**) (corresponding to deletions) for lines 11 and 12 between two target sites, for both *XylT* and *FucT*. For line 11, the amplicons for *FucT* and *XylT* were cloned into pGEM-T Easy and sequenced. In total, we retrieved 36 sequences for *FucT* and seven for *XylT*, which resulted in six different sequences for *FucT* and four for *XylT* (**Figure 3B**). The sequencing results confirmed that small INDEL’s and deletions between the target sites took place.

**FIGURE 4:**
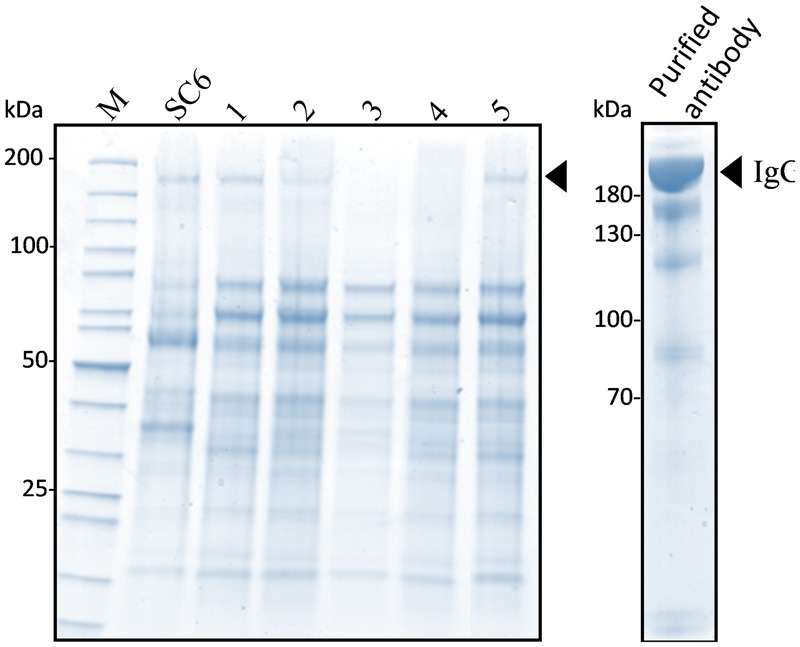
**Expression of human IgG2 in the *XylT/FucT* KO line 11.** The extracellular medium (30 μL) of five kanamycin resistant lines was analyzed by non-reducing SDS-PAGE followed by colloidal blue staining. The lane on the right hand side shows the peak elution fraction of the purified antibody. Arrowhead indicates the band corresponding to the IgG2.

The KO lines did not display any particular phenotype and grew as WT cells. To demonstrate that a KO line can be used for expressing an ectopic protein, we transformed the KO line 11 with a binary vector (pPZP-RCS2-nptII-mCherry-HIgG2-LoBM2) designed for the production of a human IgG2 antibody (Mercx et al., 2016). The expression of the antibody was checked in five kanamycin resistant lines. The extracellular medium of 7-day old cultures was analyzed by non-reducing SDS-PAGE (**Figure 4**). As a control, we also analyzed the SC6 transgenic line that had been previously obtained with the same antibody-encoding binary vector (Mercx et al., 2016). The intensity of the band corresponding to the intact antibody for lines 11/1 and 11/5 were as high as for the control SC6 transgenic line, meaning that the antibody production rate in the KO line was not affected. We then purified the antibody from line 11/5 grown in eight 50 mL-Erlenmeyer flasks for 7 days (**Figure 4**). The glycosylation profile was checked by mass spectrometry (**Table 2**). As expected, no β(1,2)-xylose or α(1,3)-fucose residues were identified on the IgG expressed in the KO line. The major structure in the KO line 11/5 was GnGn (69%). The second major structure was Man7 representing 9.3% and several other high-mannose type glycans as well as GnM were also identified.

**Table 2:** Relative amount of *N*-glycans (%) on the IgG secreted in the KO line 11/5.

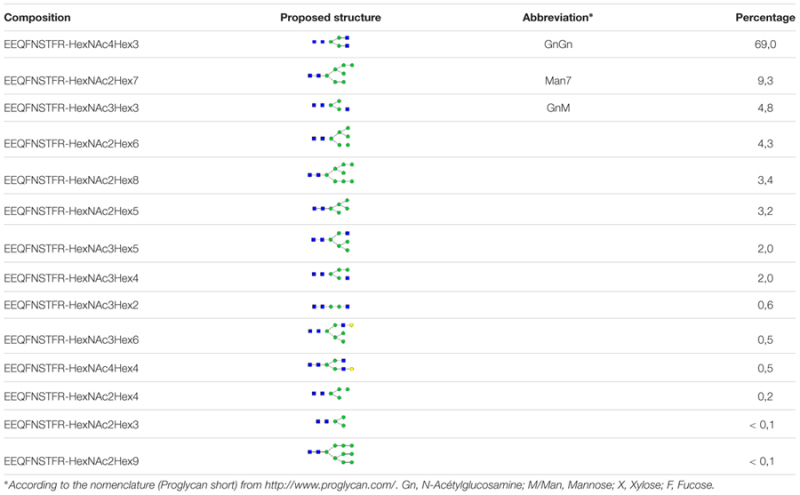

## Discussion

In this work, we took advantage of the recent development of CRISPR/Cas9 and its easiness to target multiple sequences to inactivate the *XylT* and *FucT* genes.

We identified two *XylT* and four *FucT* isoforms, and a total of 12 loci were disrupted. While this article was under review, Hanania et al. (2017) published a very similar study inactivating the β(1,2)-xylosyltransferase and the α(1,3)-fucosyltransferase genes in *N. tabacum* BY-2 cells with CRISPR/Cas9. These authors mentioned that five FucT genes were inactivated. We figured out that the isoform called *FucT-B* (Ntab-BX_AWOK-SS16887) by Hanania et al. (2017) is only present in one out of three cultivars (in BX and not in TN90 and K326) and is identical to *FucTD* (called *FucT-A* in Hanania et al., 2017) in the 5′ region and identical to *FucTC* (called *FucT-C* in Hanania et al., 2017) in the 3′ region. This gene might therefore have arisen in one cultivar by recombination or might be a sequence assembly artifact. In this study we chose three conserved regions for each target. For *XylT*, a single sgRNA per region was sufficient to target the two isoforms, but for *FucT*, we were unable to find a 20 bp conserved region near a PAM sequence for the four isoforms. Thus, two sgRNAs were designed for each target region. Editing was quite efficient since, in each sequence we retrieved from *FucT*, mutations occurred in at least two of the three targets. For *XylT*, among the four mutated sequences obtained, three were mutated in at least two target sites, and only one at a single site. The efficiency of targeting can be explained by the fact that Cas9 and the sgRNAs are stably integrated into the genome and are continuously expressed. A drawback of the CRISPR-Cas9 editing system is that, although rare, off-targets might be generated (Puchta, 2016). However, after fifteen transfers, the transgenic lines looked fine and grew similar to the WT.

Because the *N. tabacum* genome is not fully sequenced, nor annotated, we cannot rule out that a still unknown *XylT* or *FucT* gene was not targeted by the CRISPR-Cas9 approach. However, in this case its expression in BY-2 cells must be below detection since the Western blotting analysis did not show any residual signal neither for the anti-α(1,3)-fucose nor for the anti-xylose antibody. The *N*-glycans analysis by MALDI-TOF showed that lines 11 and 12 do not display detectable β(1,2)-xylose and α(1,3)-fucose unlike the WT line, for which more than 91% of the *N*-glycans carry a β(1,2)-xylose and more than 83% carry a α(1,3)-fucose residue. Several high-mannose type glycans are also present. This is in agreement with a study from Misaki et al. (2001), which showed that these high-rich mannose *N*-glycans are present on secreted proteins from *N. tabacum* BY-2 cells.

One advantage of plants compared to animal platform production is the homogeneity of glycosylation. Here we confirmed that glycosylation of the secreted proteins is quite homogenous with more than 80% of the total *N*-glycans being either GnMXF (42.4%), MMXF (31.5%), or GnGnXF (8.5%) for the WT line. Interestingly lines 11 and 12 still improved this homogeneity with more than 87% of the total *N*-glycans being either GnGn (39.9-44.6%), GnM (41.1-39.1%) or MM (6.4-4.8%).

We can point out that the paucimannosidic *N*-glycans are much less present in the KO lines (**Table 1**). This correlates with a recent study (Shin et al., 2016) that demonstrated that in *N. benthamiana*, the presence of the core α(1,3)-fucose on the *N*-glycans enhances the trimming of GlcNAc residues. These authors showed that this trimming is due to the activity of β-hexosaminidases, mainly located in the plasma membrane in leaf epidermal cells. Paucimannosidic *N*-glycans increase the heterogeneity of the therapeutic product and may affect the biological activity of the recombinant protein. Thus, by expressing a recombinant protein in these *XylT*/*FucT* KO lines we should increase the homogeneity of the glycosylation profile and reduce the unwanted truncated *N*-glycans.

The expression level of an antibody in a *XylT/FucT* KO line was similar as that in a *FucT* and *XylT* wild-type line. Knocking-out these genes is therefore not detrimental to the expression of an ectopic protein. The glycosylation profile of the IgG secreted in the *XylT*/*FucT* KO line was consistent with the profile identified for the total *N*-glycans of the secreted proteins. However, a lower amount of paucimannosidic structures was identified for the IgG compared to the total secreted proteins. This is probably because the antibody glycosylation site is little accessible for β-hexosaminidases.

Plant specific glycans, especially β(1,2)-xylose and α(1,3)-fucose, have been for years a real hurdle for the regulatory approval of recombinant proteins expressed in plants (Santos et al., 2016). Here, we provide an expression platform that possesses all of the conditions to produce recombinant proteins under GMP with no regulatory issues. Indeed, plant suspension cells can be grown in contained bioreactors similar to the current mammalian production platform, and thus do not suffer from the absence of regulatory pathways such as for whole plant production. Compared to mammalian production platforms such as Chinese hamster ovary (CHO) cells, plant cells have two main advantages: they cannot be contaminated with animal pathogens (e.g., viruses or prions) and the culture medium is particularly cheap. On the negative side, plant cells still have a lower production rate (e.g., 30–100 mg IgG/ml; Vasilev et al., 2013; Magy et al., 2014) while CHO cells can produce more than 1 g/L (Kunert and Reinhart, 2016). However, the production in plant cells has not yet benefited from the long research history that characterizes animal cells and yield improvement can be expected at different levels such as fed-batch strategies, elite line selection, gene amplification, inhibition of proteolytic activity, medium optimization (Santos et al., 2016).

Besides cell suspensions, *Agrobacterium-* or virus-mediated transient expression in leaf tissues is another interesting plant production system. It is cheap, fast and can be easily up-scaled. A production yield of 1 mg of rituximab antibody per gram leaf (fresh weight) has been reported (Diamos et al., 2016). However, down-stream processing (e.g., purification) represents an important part of the production costs which should be lower with suspension cells when the protein of interest is secreted. Indeed, in this case the protein has to be purified from the external medium which is less complex in the absence of plant fibers and a range of secondary metabolites and proteins than a total leaf extract (Yao et al., 2015). In addition, confinement and reproducibility are better controlled in stable cell suspension cultures. Now that potential immunogenic and/or allergic residues have been removed, no additional regulatory issues could prevent the emergence of plant cell-produced recombinant proteins onto the market.

## Author Contributions

SM performed the research, analyzed the data and wrote the first draft of the manuscript. NS performed the mass spectrometry analysis. CN and MB conceived and supervised the project. ED and FC supervised the project. All authors made revision to the manuscript and approved the final manuscript.

## Conflict of Interest Statement

The authors declare that the research was conducted in the absence of any commercial or financial relationships that could be construed as a potential conflict of interest.

## Footnotes

1 https://solgenomics.net/organism/Nicotiana_tabacum/genome
