# Supplementary material for: Inactivation of the β(1,2)-xylosyltransferase and the α(1,3)-fucosyltransferase genes in Nicotiana tabacum BY-2 Cells by a Multiplex CRISPR/Cas9 Strategy Results in Glycoproteins without Plant-Specific Glycans
Source: Front Plant Sci. 2017 Mar 27;8:403. doi: 10.3389/fpls.2017.00403 (PMC5366340; doi:10.3389/fpls.2017.00403)
Supplement: Supplementary file 1 [file Table_1.DOCX]

| Table S1. Primers used in this study | |
| --- | --- |
| Primers name | Sequences (5' to 3') |
| FUCT-Fwd | CACGACGAAGGGGATATGAT |
| FUCT-Rev | TCATTAGAATTCTCAAAAGC |
| XYLT-Fwd | ATGAACAAGAAAAAGCTGAAA |
| XYLT-Rev | ACTCCTACCAATAACCGACT |
