# Supplementary figures and images for: Inactivation of the β(1,2)-xylosyltransferase and the α(1,3)-fucosyltransferase genes in Nicotiana tabacum BY-2 Cells by a Multiplex CRISPR/Cas9 Strategy Results in Glycoproteins without Plant-Specific Glycans
Source: Front Plant Sci. 2017 Mar 27;8:403. doi: 10.3389/fpls.2017.00403 (PMC5366340; doi:10.3389/fpls.2017.00403)

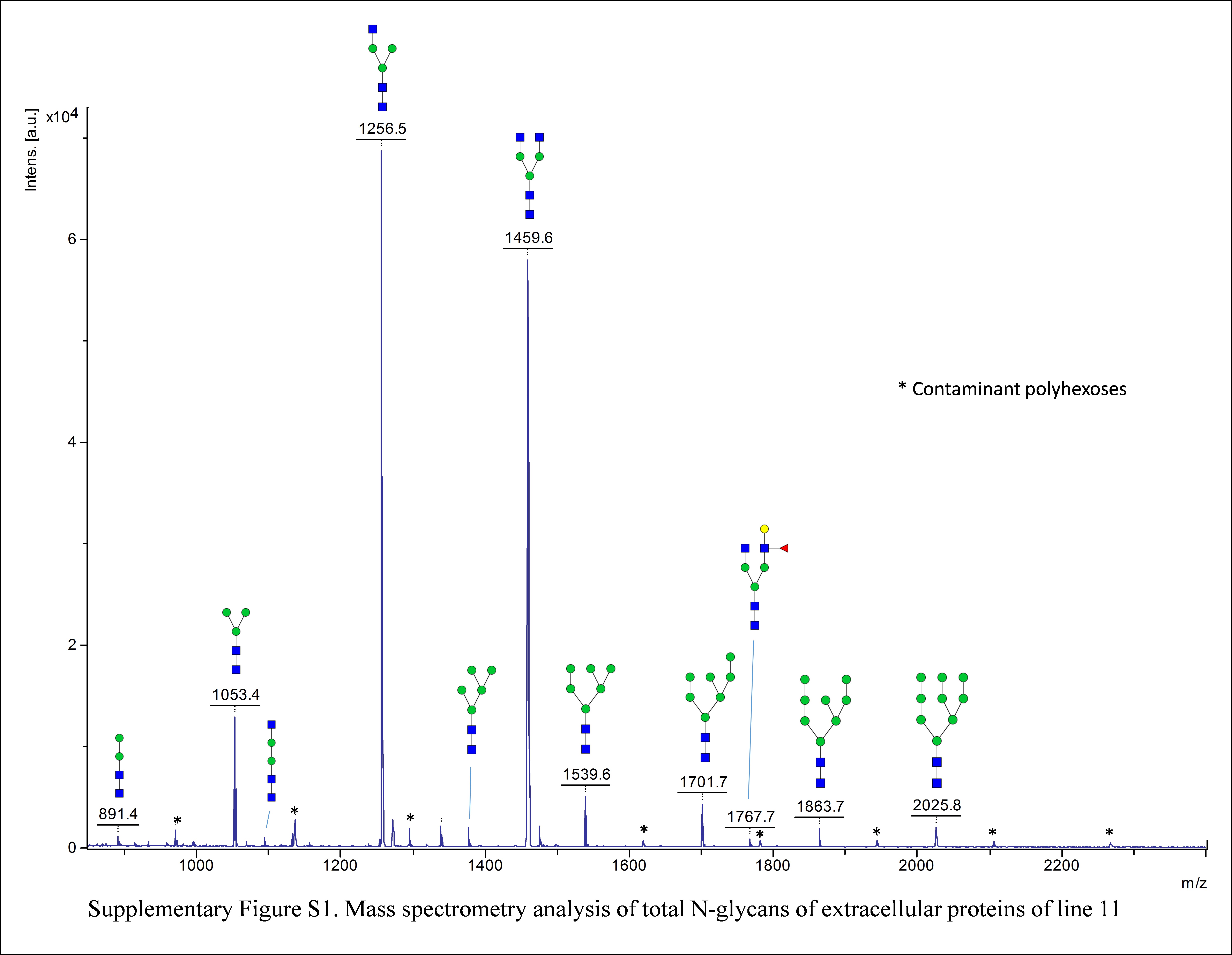

Supplement: Supplementary file 2 [file Image_1.jpg]

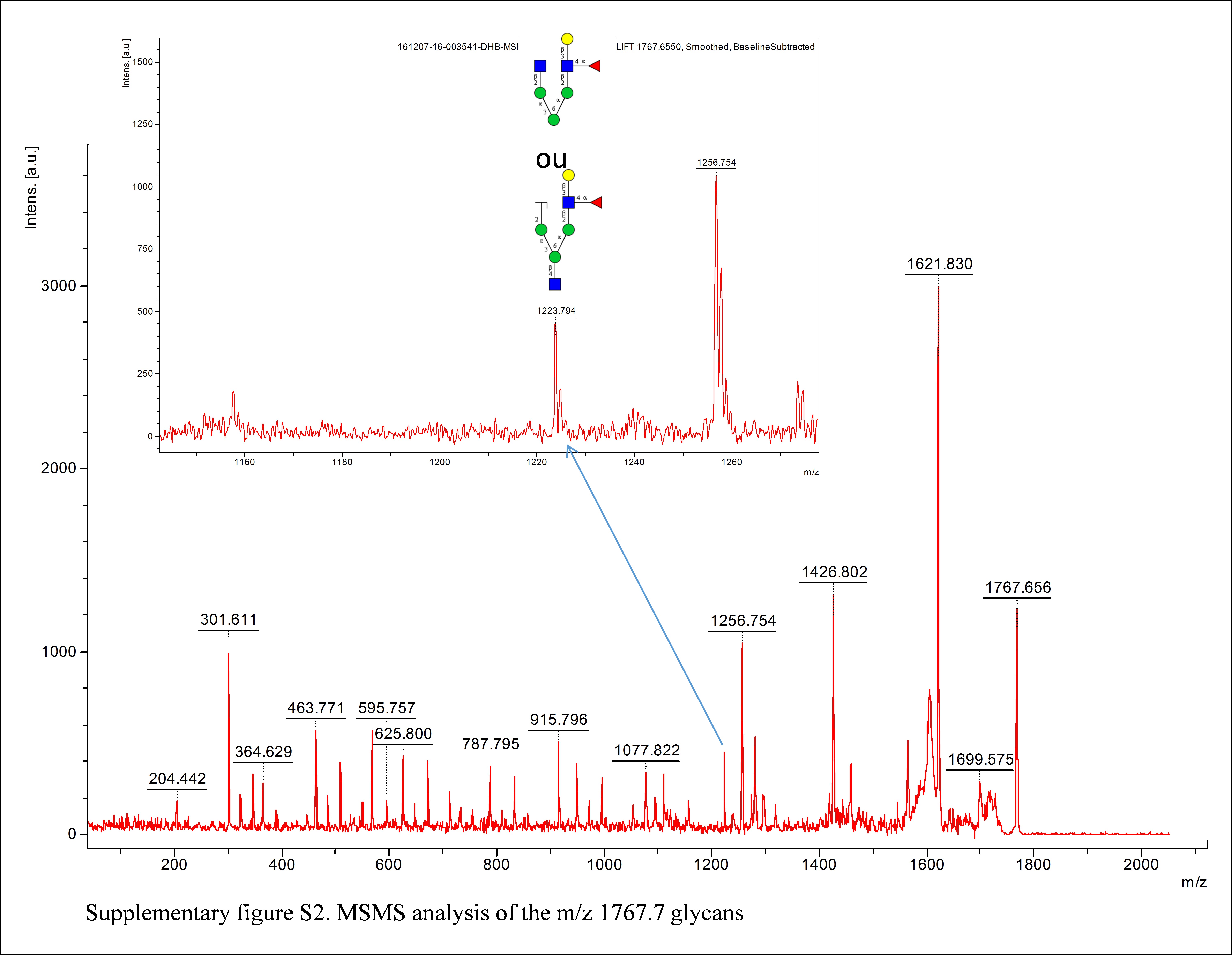

Supplement: Supplementary file 3 [file Image_2.jpg]

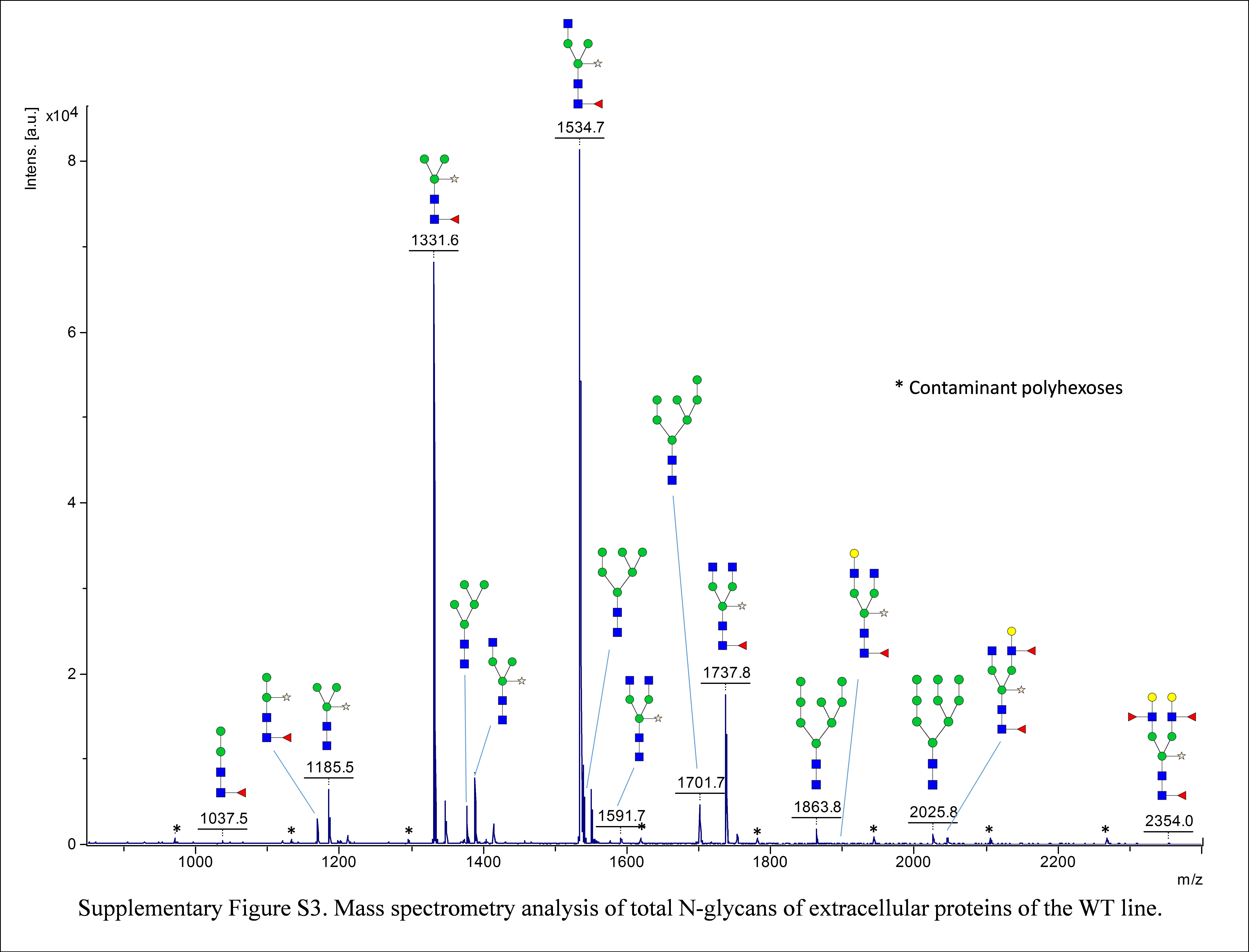

Supplement: Supplementary file 4 [file Image_3.jpg]
